# Ant-like Traits in Wingless Parasitoids Repel Attack from Wolf Spiders

**DOI:** 10.1007/s10886-018-0989-2

**Published:** 2018-07-31

**Authors:** Jeffrey A. Harvey, Bertanne Visser, Marl Lammers, Janine Marien, Jonathan Gershenzon, Paul J. Ode, Robin Heinen, Rieta Gols, Jacintha Ellers

**Affiliations:** 1 Department of Terrestrial Ecology, Netherlands Institute of Ecology, Droevendaalsesteeg 10, 6700 AB Wageningen, The Netherlands; 2 Department of Ecological Sciences, Section Animal Ecology, VU University Amsterdam, De Boelelaan 1085, 1081 HV Amsterdam, The Netherlands; 3 Evolutionary Ecology and Genetics group, Biodiversity Research Centre, Earth and Life Institute, Université Catholique de Louvain, Croix du Sud 4-5, 1348 Louvain-la-Neuve, Belgium; 4 Max Planck Institute of Chemical Ecology, Beutenberg Campus, Hans Knoel Str 8, DE-07745 Jena, Germany; 5 Department of Bioagricultural Sciences and Pest Management, Colorado State University, Fort Collins, CO 80523-1177 USA; 6 Laboratory of Entomology, Wageningen University, Droevendaalsesteeg 1, 6700 EH Wageningen, the Netherlands

**Keywords:** *Lasius*, *Formica*, *Gelis*, Hymenoptera, Predation, Chemical defense, Batesian mimicry; Müllerian mimicry

## Abstract

A recent study showed that a wingless parasitoid, *Gelis agilis*, exhibits a suite of ant-like traits that repels attack from wolf spiders. When agitated, *G. agilis* secreted 6-methyl-5-hepten-2-one (sulcatone), which a small number of ant species produce as an alarm/panic pheromone. Here, we tested four *Gelis* parasitoid species, occurring in the same food chain and microhabitats, for the presence of sulcatone and conducted two-species choice bioassays with wolf spiders to determine their degree of susceptibility to attack. All four *Gelis* species, including both winged and wingless species, produced sulcatone, whereas a closely related species, *Acrolyta nens*, and the more distantly related *Cotesia glomerata,* did not. In two-choice bioassays, spiders overwhelmingly rejected the wingless *Gelis* species, preferring *A. nens* and *C. glomerata*. However, spiders exhibited no preference for either *A. nens* or *G. areator*, both of which are winged. Wingless gelines exhibited several ant-like traits, perhaps accounting for the reluctance of spiders to attack them. On the other hand, despite producing sulcatone, the winged *G. areator* more closely resembles other winged cryptines like *A. nens*, making it harder for spiders to distinguish between these two species. *C. glomerata* was also preferred by spiders over *A. nens*, suggesting that other non-sulcatone producing cryptines nevertheless possess traits that make them less attractive as prey. Phylogenetic reconstruction of the Cryptinae reveals that *G. hortensis* and *G. proximus* are ‘sister’species, with *G. agilis*, and *G.areator* in particular evolving along more distant trajectories. We discuss the possibility that wingless *Gelis* species have evolved a suite of ant-like traits as a form, of mimicry to repel predators on the ground.

## Introduction

To eat or be eaten; that is one of the major paradigms among predators and their prey in ecology. In a co-evolutionary framework, predators evolve adaptations that enable them to locate, subdue, and consume their prey more successfully, whereas their potential victims have evolved a suite of defenses to avoid, escape, or resist attack. Among insects, selection imposed by predators has led to a staggering array of adaptations that reduce prey susceptibility. For example, some species seek habitats where they are less prone to attack from natural enemies, a process known as ‘enemy-free-space’ (Jeffries and Lawton 1984; Mulatu et al. 2004; Stamp 2001). Many invertebrates are also cryptically colored and blend into the background of the habitat in which they are found, making it hard for visually foraging predators to locate them (Endler 1981; Starrett 1993). Crypsis often involves resemblance to natural structures, such as bird feces, lichens, leaves, twigs and stones (Skelhorn et al. 2010). Other invertebrates defend themselves by exhibiting aggressive responses to attackers (Gentry and Dyer 2002; Greeney et al. 2012; Gross 1993) or by possessing physical characteristics, such as spines, hairs, a thickened cuticle or other structures that render them less susceptible to attack or reduce palatability (Dahl and Peckarsky 2003; Dyer 1997; Gross 1993).

Chemical defenses are also widely employed by many invertebrates in nature as defenses against their antagonists (Rowell-Rahier et al. 1995; Zvereva and Kozlov 2016). These chemicals can be internally synthesized and then deployed directly against an attacker, as in ants, cockroaches, and bombardier beetles (Baldwin et al. 1990; Eisner and Aneshansley 1999; Rossini et al. 1997). When discharged their main function is to temporarily disable or dissuade attackers. Alternatively, toxic chemicals are metabolized and stored in body tissues and advertised via bright aposematic warning coloration (Mallet and Joron 1999; Marples et al. 1994; Opitz and Müller 2009), and are toxic when ingested by another organism. In the latter case, the toxins are perceived through visual detection by predators. However, a third possibility is that chemicals are not physically discharged, but are passively released onto the cuticle of an organism and are detected by potential attackers either through smell or taste.

Many species in nature have evolved defensive traits that resemble traits expressed by other organisms and function to reduce susceptibility to predators or parasitoids. This resemblance is not necessarily an example of evolved mimicry, but may be incidental. For instance, many highly palatable flies, moths and other insects have evolved a striking yellow-black body coloration that closely mimics the body coloration of stinging species such as bees and wasps and hence are avoided by potential predators (Quicke 2017). Moreover, late instar larvae of some butterflies and moths of several lepidopteran families (e.g., Papilionidae, Sphingidae, Lepidptera) possess large ‘eye-spots’ just behind the head capsule that closely resemble the eyes of snakes. These eye-spots may drive away potential predators because the caterpillar itself appears to be a predator (Janzen et al. 2010; Hossie and Sherratt 2012). Alternatively, the eye-spots may be an example of sexual selection, with males possessing larger or more symmetrical eyespots being more attractive to females and thus enhancing their fitness (Oliver et al. 2009; Monteiro 2015). In this way, the defensive benefit of these eyespots may be simply a secondary function. It is important to exercise caution when attributing an evolutionary explanation to a trait that may instead have arisen for an altogether different reason.

Amongst parasitoid wasps (Hymenoptera), the genus *Gelis* (Ichneumonidae) is well very well represented in the Palearctic with many species found across the region (Schwarz and Shaw 1999). They are found in a variety of habitats (e.g., fields, forest margins, even in trees), although a few species are also fully winged (Visser et al. 2014, 2016). Although their ecology and host ranges are poorly studied, *Gelis* species are considered to be highly generalist primary and secondary parasitoids (hyperparasitoids), attacking hosts as phylogenetically diverse as spider egg sacs, moth pupae, and parasitoid cocoons (Cobb and Cobb 2004; Fitton et al. 1987; Harvey 2008; Schwarz and Boriani 1994; Wieber et al. 1995). In central Europe, several wingless *Gelis* species are abundant at forest edges and grassy meadows (Harvey et al. 2014).

Many *Gelis* species are wingless and closely resemble ants morphologically and even chemically. For example, Malcicka et al. (2015) found that *Gelis agilis* Fabricius (Hymenoptera: Ichneumonidae, Cryptinae) closely resembles the common black ant *Lasius fuliginosus* Latreille (Hymenoptera: Formicidae) in terms of general body shape and size, color and also defensive chemistry. When agitated, both *G. agilis* and *L. fuliginosus* secrete 6-methyl-5-hepten-2-one (sulcatone) that functions as an alarm/panic pheromone. In *G. agilis*, sulcatone is apparently secreted from a gland in the head capsule, is highly volatile, and adheres to the cuticle of the wasp. A study by Malcicka et al. (2015) found that both *G. agilis* and the common black ant *Lasius niger* L. (Hymenoptera: Formicidae) were almost never attacked by spiders in arenas. Many ants are predators and are considered important agents of selection in temperate and tropical habitats across the world (Hölldobler and Wilson 1990). Because ants nest in colonies that many contain thousands of individuals, they are often studiously avoided by other arthropods living in the vicinity of their nests. Under these conditions, it is not surprising that *Gelis* species exhibiting similar traits may benefit by being better able to escape from or repel their own natural enemies that live in the same habitats.

The close chemical and morphological similarity between parasitoids and ants has thus far only been studied in one *Gelis* species, *G. agilis*. We, therefore, do not know if these traits are found in other *Gelis* species or in non-congeneric species within the same family and subfamily (Ichneumonidae, Cryptinae). Adopting a comparative approach, the current study thus aims to (1) compare ant-like traits in three other *Gelis* species, including the winged *G. areator* Panzer, and two wingless species, *G. hortensis* Christ and *G. proximus* Forster focusing on morphological similarities and the production of sulcatone; (2) determine if sulcatone is produced by the phylogenetically-close, winged species *Acrolyta nens* Hartig (Hymenoptera: Ichneumonidae, Cryptinae) and a more distantly related species, *Cotesia glomerata* L. (Hymenoptera: Braconidae, Microgastrinae); and (3) measure feeding preferences of wolf spiders in dual-species choice bioassays. We argue that the expression of ant-like traits in wingless *Gelis* species might be a form of ant mimicry (myrmecomorphy) and show that sulcatone in particular acts as a putative defense against cursorial predators like wolf spiders.

## Methods and Materials

### Insects and Spiders

All insects were reared at a temperature of 22 ± 2 °C under a 16:8 h L:D regime. Cultures of the parasitoid *C. glomerata* and its host, the large cabbage white butterfly *P. brassicae* were obtained from insects reared at Wageningen University (WUR), the Netherlands, and were collected from agricultural fields in the vicinity of the University. All *P. brassicae* larvae used in these experiments had been maintained on *Brassica oleracea* var. Cyrus (Brussels sprouts) at WUR.

*Cotesia glomerata* L. (Hymenoptera: Braconidae) typically oviposits 10–40 eggs into first (L1) to third (L3) instars of *P. brassicae*. During their development, the parasitoid larvae feed primarily on host hemolymph and fat body. Fully grown larvae emerge from the host caterpillar late during its final instar, and spin cocoons adjacent to the host, which perishes within a few days. Once weekly, several hundred L2 *P. brassicae* were presented to mated female *C. glomerata* in rearing cages (30 × 30 × 30 cm) for parasitism. Parasitized caterpillars were then transferred to steel and plexiglass cages (30 × 30 × 60 cm) containing cabbage plants. Fresh parasitoid cocoons were collected from these cages.

*Gelis agilis*, *G. proximus*, *G. hortensis*, *G. areator* and *A. nens* were collected by pinning cocoons of *C. glomerata* onto the shoots of black mustard (*Brassica nigra*) or garlic mustard (*Alliaria petiolata*) plants or placed directly onto the ground adjacent to mustard stems in a grassy field margin adjacent to the Netherlands Institute of Ecology (Wageningen, the Netherlands). In culture, the five hyperparasitoids were maintained exclusively on fresh cocoons of *C. glomerata*. After emergence, each species was separately kept in closed, meshed rearing cages (30 × 30 × 30 cm) with honey and water and stored at 10 ± 1 °C in incubators.

Wolf spiders from several genera (e.g., *Pardosa*, *Alopecosa*, *Arctosa*) were collected by hand from field margins adjacent to the Netherlands Institute of Ecology (Wageningen, the Netherlands). Spiders were placed individually in Petri dishes (8 cm diam.) with water absorbed into a cotton ball and kept at a temperature of 22 ± 2 °C in a climate room.

### Parasitoid-Spider Bioassays

Although both sexes of wingless *Gelis* species are very ant-like in appearance, only male wasps were used in these experiments, because they are produced in much greater numbers in our cultures. To test whether closely related geline species were repellent to spiders, spiders were kept without food (prey) for several days prior to dual-choice assays to increase their hunger level. For assays, spiders were individually transferred to large Petri dishes (12 cm diam.) containing water absorbed into a cotton ball. Two *C. glomerata* males were placed into the dish along with two geline males of the same species. Dishes were then monitored over the course of several hours for predation, with the first parasitoid species to be attacked recorded. Dishes were then left for 24 h and if any spiders had still not attacked any prey after that time wasps were removed. Prey preference was based only on spiders that attacked prey during the 24 h period. During several assays, however, more than a single prey was attacked. In almost every instance they were the same species; e.g., *C. glomerata*. If we could not ascertain which species was attacked first, the data from that Petri dish was excluded. The experiment was repeated with different individual spiders using two males of each species in the following combinations: *G. proximus* – *A. nens*; *G. hortensis* – *C. glomerata*; *G. hortensis* – *A. nens*; *G. areator* – *C. glomerata*; *G. areator* – *A. nens*; *A. nens* – *C. glomerata*. Spiders were only used once. Following assays, spiders were released back into the field.

### Chemical Analyses of Parasitoids

Chemical analysis took place at the Max Planck Institute, Jena, Germany. For initial analysis, volatile chemical releases of all species were determined using an APCI-MS. Five adult males of each species tested were agitated by pinching them with soft forceps and then separately placed in 20 ml glass scintillation vials. The wingless *Gelis* species – but none the other parasitoids tested here - produce a very distinctive and pungent odor when they are agitated. The APCI-MS sampling point draws in a continuous air stream at 25 ml min − 1, into a heated transfer line (~160 °C) through a deactivated silica tube (1 m × 0.53 mm ID) before entering the APCI source. Volatiles entering the source were ionized by a positive ion corona discharge (4 kV), which typically forms the adduct ion M + H+. Spectra were recorded using a Platform II mass spectrometer across a mass range of 25–250 Da, with the cone voltage set to 18 V. Two major ions with the m/z of 108 and 127, respectively, were observed, consistent with the fragmentation pattern of an unsaturated terpenoid with a molecular mass of *M* = 126 (127). To confirm the identity of the chemical released, five males of each species were separately placed in a 20 ml flask under the same protocol as the APCI analysis. Flasks were then sealed with a polytetrafluoroethylene-lined septum. Volatile compounds were transferred for GC-MS analysis using a SPME fibre (50/30 mm, assembly Divinylbenzene/Carboxen/Polydimethylsiloxane, (Supelco, Bellefonte, PA, USA), which was exposed in the flask headspace for 10 min at 22 °C. Desorption of volatile compounds attached to the fibre occurred in the injector of an Agilent 6890 Gas chromatograph coupled to an Agilent 5973 mass spectrometer (Agilent Technologies, Waldbronn, Germany) at 250 °C Volatile compounds were separated on a DB5MS column (DB5MS, 30 m × 0.25 mm × 0.25 μm film, Agilent Technologies, Waldbronn, Germany)). The chromatographic conditions were: splitless injection, initial oven temperature, 40 °C for 1 min, increased at 8 °C/min to 120 °C followed by an increase of 60 °C/min to 300 °C and hold for 2 min. Parameters of the mass spectrometer for electron impact sample ionization were as follows: interface temperature, 270 °C; repeller, 30 V; emission, 34.6 μA; electron energy, 70 eV; source temperature, 230 °C. Mass spectrometer was run in scan mode in the mass range m/z 33 to 350. For identification of sulcatone (6-methyl-5-hepten-2-one) the mass spectrum of the peak with a retention time of ~8.2 min was compared to the entry of sulcatone in the commercially available Wiley mass spectra library (see also methods for identification of sulcatone in Malcicka et al. 2015). Moreover, spectral comparisons with published literature indicated a consistency with sulcatone.

### Reconstructing Partial Phylogenetic Tree of the Cryptinae

Whole-body DNA was isolated from *Gelis agilis* (*n* = 3), *Gelis areator* (*n* = 3), *Gelis hortensis* (*n* = 3), *Gelis proximus* (*n* = 3), and *Acrolyta nens* (*n* = 4). Voucher specimens from the same cultures are stored at the Department of Ecological Science at the Vrije Universiteit Amsterdam under numbers ML.V001.001-ML.V001.015. Before DNA isolation the individual wasps were washed in 70% ethanol and dried under vacuum. Animals were crushed in 100 μL PBS and the tissue was lysed by adding 100 μL Nuclei Lysis Solution (Promega) and 2 μl Proteinase K (Roche), vortexed and incubated for 15 min at 65 °C. Further tissue lysis was achieved by adding 170 μL DNA lysis buffer (Promega). The lysates were centrifuged for 10 min at full speed and the DNA was retrieved from the supernatant using Promega DNA spin columns. Extracted DNA was eluted in 50 μL H_2_O.

To amplify ±700 bp of the COI gene a newly developed degenerate primer set was used that worked on three *Gelis* species. For *Gelis areator* a more specific primer set was developed and used these for *Acrolyta nens* as well. All primer sequences are listed in Table 1. We performed the Promega GOTaq DNA Polymerase protocol for all samples and additionally added *pfu* polymerase (Promega) for proofreading. All components of the 35-cycle PCR reactions are listed in Table 2. Input DNA template was 1 μL giving a final reaction volume of 25 μL.

**Table 1:** Overview of PCR primers used in this study

Primer set name	Forward primer name	Forward primer sequence	Reverse primer name	Reverse primer sequence	Annealing temperature
*Gelis* COI universal	Ga_COI-uniF	TCAACMAATCATAAAGATATTGG	Ga_COI-uniR	TAAACTTCWGGRTGWCCAAAAAATC	48 °C
*G. aerator COI*	G.are_COI-F	CATTTTTGGTATATGAGCAGG	G.are_COI-R	GGTGTTGGTATAAAATTGGATC	50 °C
pGEM-T	T7 universal	TAATACGACTCACTATAGGG	Sp6 universal	CATACGATTTAGGTGACACTATAG	55 °C

**Table 2:** Components of all PCR reactions

Compound	Volume
5× GO-taq buffer	5,0 μL
MgCl_2_ (25 mM)	1,5 μL
dNTP (2,5 mM each)	2,0 μL
Primer F (5 μM)	1,0 μL
Primer R (5 μM)	1,0 μL
GO-taq DNA polymerase	0,2 μL
*Pfu* polymerase (10%)	0,02 μL
H_2_O	13,3 μL

After cleanup of the PCR amplicons with the Wizard SV PCR and Gel Clean-up System (Promega) the fragments were ligated into pGEM-T plasmids (Promega) and transformed E.coli XL-I Blue (Stratagene) following a heat shock protocol. Positive colonies were screened by PCR with an universal T7 and Sp6 primer set (Table 1). The positive colonies were cultured overnight in 4 mL LB medium and plasmids were isolated with the Wizard plus SV Minipreps DNA Purification System (Promega) following the manufacturer’s protocol.

Miniprep samples were diluted to 100 ng/μL and sent for Sanger sequencing (with T7 or Sp6 universal primers) to MWG Eurofins. COI amplicons of *A*. *nens* were sequenced directly, using a 5 ng/μL sample with the G.are_COI-F primer added. Forward and reverse reads were trimmed using Vector NTI software package (version 11). DNA sequences are available in NCBI GenBank under accession numbers MF375801-MF375811.

*Gelis* and *Acrolyta* COI barcodes were aligned manually in Mesquite v3.04, which is trivial for these COI barcodes as there are no gaps nor indels. For each sequenced *Gelis* species the top 40 BLAST hits were downloaded from GenBank using Mesquite’s Top BLAST Matches function with default settings. This ensured that all COI sequences of related *Gelis* species were included in the analysis. Subsequently all identical sequences were removed. *Diplazon laetatorius* (Hymenoptera: Ichneumonidae: Diplazontinae) was included as outgroup. The resulting alignment consisted of 106 COI barcodes and a length of 707 base pairs. This alignment was exported in PHYLIP format for RaxML (Stamatakis 2006) and uploaded to the CIPRES Science Gateway v3.3 on phylo.org (Miller et al. 2010). RAxML was called as follows: raxmlHPC-HYBRID -T 4 -n 106GelisCOI.phy_2.result -s infile.txt -m GTRGAMMA -p 12345 -k -f a -N 1000 -× 12,345 -o KP750191_Diplazon_laetatorius_isolate_13DIP001. The output bipartitions file was formatted in FigTree v1.4.2 and InkScape v0.91 and is shown in Fig. 3. It should be noted that the aim here was to place the findings from the bio-assays and chemical profiles in a phylogenetic context for which a COI-barcode tree suffices, not to fully review the phylogenetic relationships of the subfamily Cryptinae. That would require more markers and a thorough taxon sampling.

### Statistical Analysis

The spider feeding choice assays were statistically analyzed by using binomial tests (z-tests) for each of the following combinations; *G. proximus* – *A. nens* (*n* = 18); *G. proximus* - *C. glomerata*; (*n* = 18); *G. hortensis* – *C. glomerata* (*n* = 21); *G. hortensis* – *A. nens* (*n* = 23); *G. areator* – *C. glomerata* (*n* = 21); *G. areator* – *A. nens* (*n* = 16); *A. nens* – *C. glomerata* (*n* = 24). The binomial test was used to test if the percentage of ‘successes’ for each combination (e.g. the species of parasaitoid first attacked by wolf spiders in the Petri dishes) significantly differed from the given probability, which was set to 0.5 (i.e. the no preference scenario). Analyses were subsequently performed using the binomial test function in R (R Development Core Team 2008).

## Results

### Ant-like Traits in *Gelis* Species

**Fig. 1:**
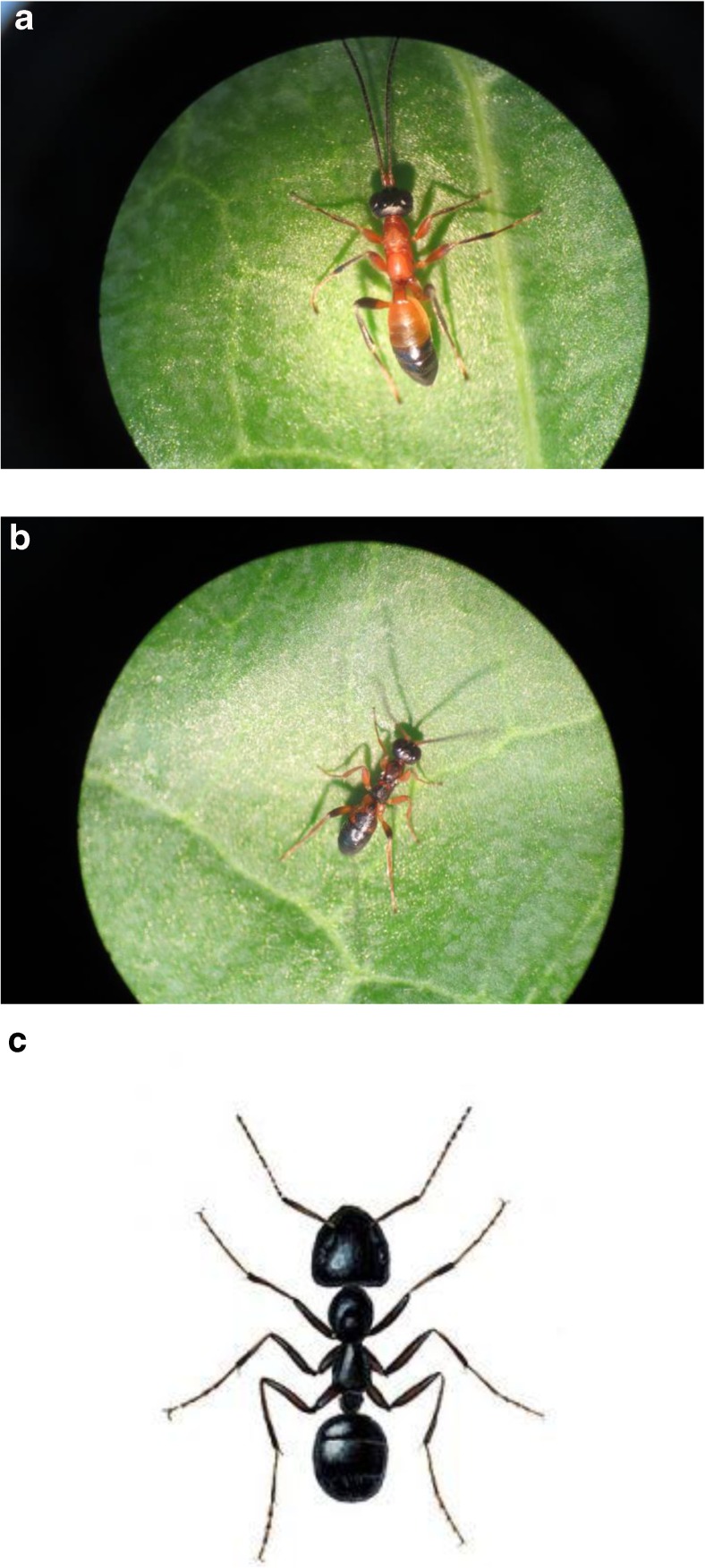
Photographs of male *Gelis hortenis* (top), *G. proximus* (middle) and a worker of the sympatric ant *Lasius fuliginosus* (bottom) showing morphological similarity of the gelines to ants. Moreover, when threatened, all three species secrete 6-methyl-5-hepten-2-one (sulcatone) as a defensive alarm/panic pheromone

The wingless *Gelis* species exhibit strong morphological similarity to ants in the genus *Lasius* (Fig. 1). Three ant species (*L. niger*, *L. flavus* and *L. fuliginosus*) are abundant across much of Europe and are found in the same local habitats at various spatial scales as the wingless *Gelis* species studied here.

### Spider Bioassays

**Fig. 2:**
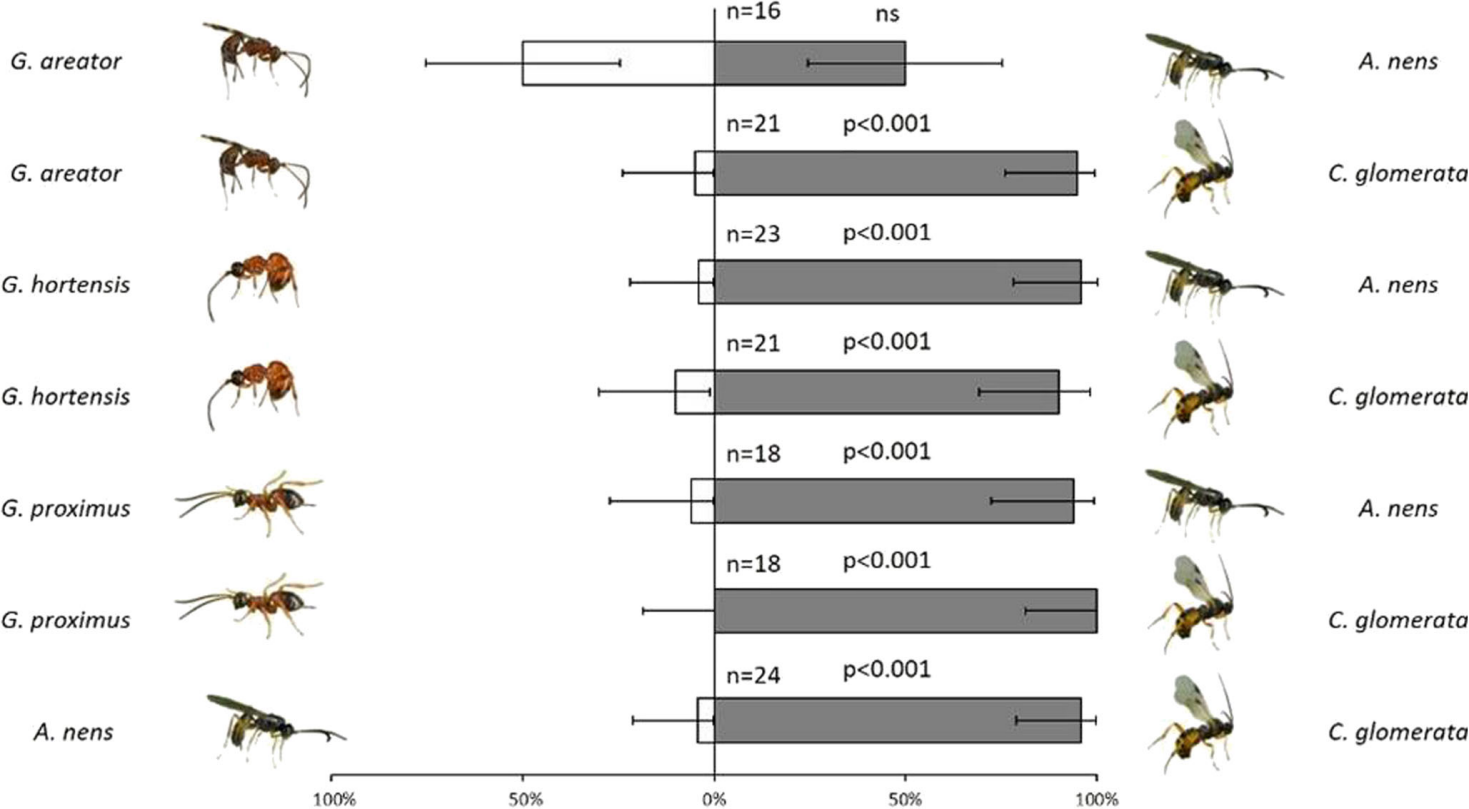
Result of dual choice assays with wolf spiders for parasitoids and hyperparasitoids used in this study. Shaded section of the bars indicate percentage of the species of parasitoid that was attacked first by wolf spiders in the bioassays. Line bars represent 95% confidence intervals. Statistical significance of the results (*P*-values) are shown beside each two species choice

In 6 of the 7 species choice assays binomial analyses revealed that spiders exhibited a highly significant preference for one species. *G. proximus* – *C. glomerata*, *z* = 4.01, *P* < 0.0001; *G. proximus* – *A. nens*, *z* = 3.54, *P* < 0.0001; *G. hortensis* – *C. glomerata*, *z* = 3.49, *P* < 0.0001; *G. hortensis* – *A. nens*, *z* = 4.17, *P* < 0.0001; *A. nens* – *C. glomerata*, *z* = 4.29, *P* < 0.0001. Both wingless *Gelis* species (*G. proximus*, *G. hortensis*) were almost completely ignored by the wolf spiders which instead fed on *C. glomerata* or *A. nens* (Fig. 2). For instance, out of 80 prey attacked by spiders involving a choice between either *G. proximus*, *G. hortensis* and the other two parasitoids, 76 (95%) chose either *C. glomerata* or *A. nens*. *Cotesia glomerata* was also highly preferred over the winged *G. areator* in dual choice assays: *G. areator* – *C. glomerata*, *z* = 3.93, *P* < 0.0001. However, spiders exhibited no preference at all in assays with *G. areator* and *A. nens*: *z* = 0, *P* = 0.5.

### Chemical analyses of *Gelis* Species, *Cotesia glomerata* and *Acrolyta nens*

**Fig. 3:**
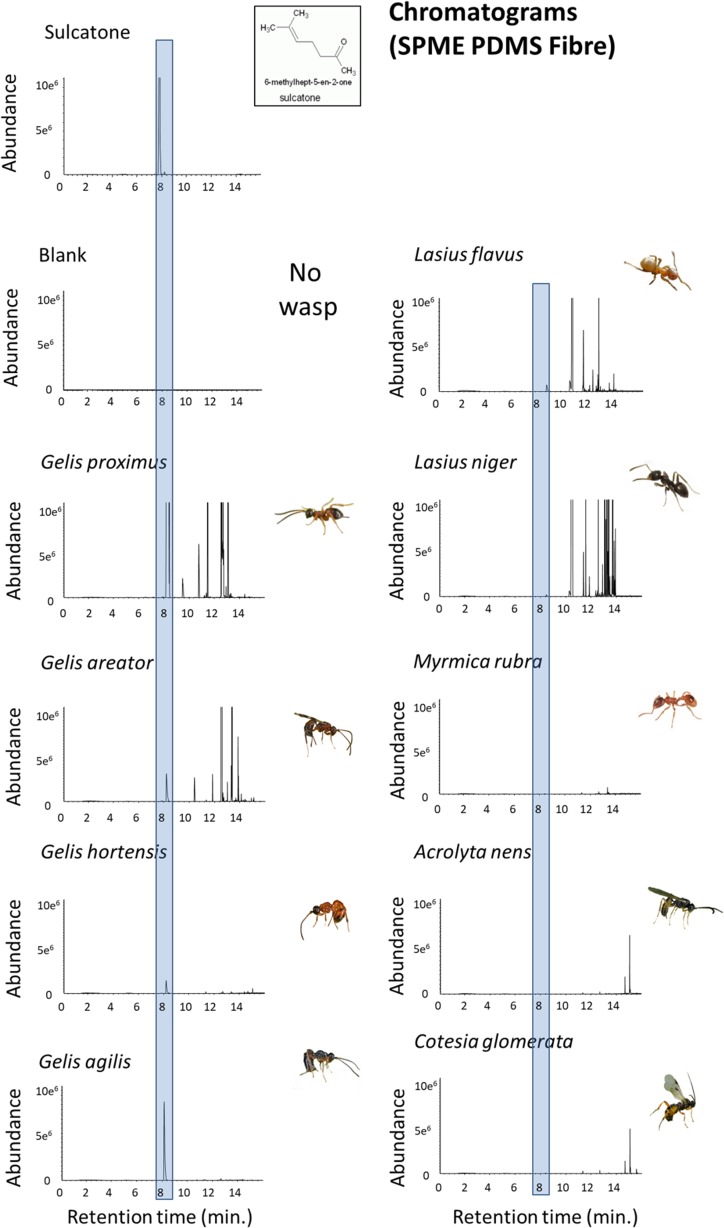
GC-MS depiction of volatile emissions including standard. GC-MS chromatogram of the main peak observed during agitation of different species of parasitoids and ants (*n* = 5 individuals per chamber). GC-MS chromatogram main peak observed during analysis of a prepared 6-methyl-5-hepten-2-one standard. The standard peak is marginally to the left of the peak shown in the gelines and ants because of recalibration

The HPMS analyses reveal that 6-methyl-5-hepten-2-one (sulcatone) was detected in assays of all four *Gelis* species tested, as well as in the two *Lasius* species. However, it was absent in the parasitoids *C. glomerata* and *A. nens* and the ant *Myrmica rubra* (Fig. 3).

### Phylogenetic Relationships of *Gelis* Species

**Fig. 4:**
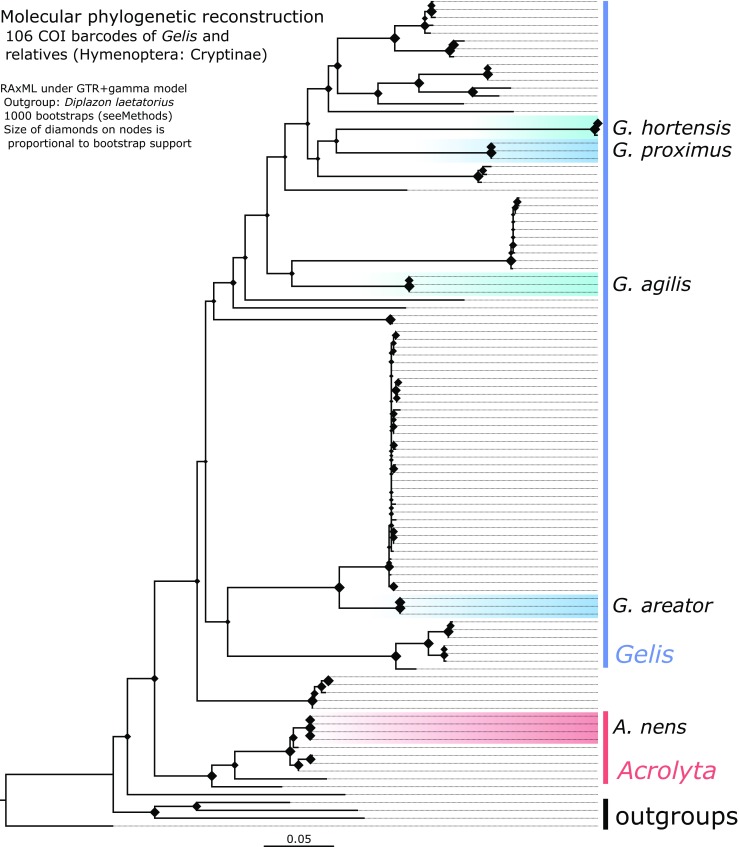
Phylogenetic reconstruction under maximum likelood criterion of geline COI barcodes in RAxML under GTR + GAMMA model with 1000 bootstraps and *Diplazon laetatorius* set as outgroup. Branch labels denote bootstrap support for the corresponding node. Newly generated barcodes from out study for *Gelis agilis*, *G*. *areator*, *G. proximus*, *G*. *hortensis* and *Acrolyta nens* are highlighted

The reconstructed COI barcode tree is shown in Fig. 4. There are no disproportionally long branches, indicating that the taxon sampling is balanced and the model underlying the analysis is appropriate. Each species currently has no other COI barcodes available in public data bases, thus our barcodes are the first published sequences of these species. Each of the five sequenced species is monophyletic in this data set with bootstrap support of 100 (i.e., maximum support). Deeper nodes in the tree have lower support, as is expected for a COI barcode tree for a species-rich genus as *Gelis*. All our *Gelis* samples are nested in a large clade of other *Gelis*-identified specimens. That suggests that *Gelis* as currently recognised forms a monophyletic clade, although it should be noted that full verification of species delimitation and genus boundaries is beyond the scope of this study.

Given the current alignment, *G*. *hortensis* and *G. proximus* are inferred as sister species. *Gelis agilis* is found sister to *G. fuscicornis*. *G*. *areator* is recovered sister to an unidentified *Gelis* species from Canada for which a lot of barcodes are available. *Acrolyta nens* is placed in a clade with sequences of another *Acrolyta* sp. This clade includes some unidentified Cryptinae and has good support (bootstrap is 93).

## Discussion

A recent study by Malcicka et al. (2015) found that the wingless facultative hyperparasitoid, *Gelis agilis* closely resembles ant species in the genus *Lasius* in two distinct ways. First, its general morphology and body color is very similar, and second, both *G. agilis* and several *Lasius* spp. secrete sulcatone when they are agitated. In ants, secretion of sulcatone by an attacked worker is detected by other workers in the colony that come to the aid of the victim, or else is perceived by the attacker that it will soon become the victim itself from other workers defending their nest-mate. In *G. agilis,* sulcatone may function in the same way and thus ‘fools’ predators, such as wolf spiders, that are abundant in the same habitats as *G. agilis*, or else it is distasteful and makes the wasps unpalatable prey. Here, we found that two other wingless geline parasitoids, *G. hortensis* and *G. proximus*, also both secreted sulcatone when they were agitated whereas the fully-winged *Cotesia glomerata* and *Acrolyta nens* did not. In dual-choice bioassays wolf spiders overwhelmingly preferred *C. glomerata* and *A. nens* over the wingless gelines. We frequently observed spiders physically contacting *Gelis* wasps with their palps in the arenas, but they were reluctant to attack and even appeared to be repelled by them. Moreover, *C. glomerata* and *A. nens* were much more active than *Gelis*, and we anticipated that they would therefore be harder prey to catch by the spiders, but this was clearly not the case.

Sulcatone produces a pungent odor that is detected in human olfactory assays at close range. Both *Lasius* species studied here secreted sulcatone whereas *M. rubra* did not. Studies with ants that excrete chemicals, including sulcatone, have shown they generate dispersal behavior in a number of predatory arthropods, and that these cues may even be used by other organisms when foraging. For example, Mestre et al. (2014) found that chemical cues from ants, including *L. niger* which produces sulcatone, induced dispersal in both a sedentary web-building spider and an active hunting spider. Moreover, Hübner and Dettner (2000) showed that the aphid hyperparasitoid *Alloxysta brevis* released secretions from the mandibles that repelled attack from web-building and jumping spiders. Halaj et al. (1997) reported that the abundance of ants in Douglas fir canopies in western Oregon was negatively correlated with spider abundance, suggesting interference competition that may also be partially chemically mediated, although the authors did not examine the mechanisms underpinning these differences.

Other studies with ants and other organisms report similar findings. McCann et al. (2013) found that red-throated caracaras, specialist predators of social wasps, incidentally acquire sulcatone on their talons when perching on trees inhabited by *Azteca* ants that produce this compound. The odor emanating from their talons is then detected by ground-nesting wasps when the caracaras perch next to the nest and leads to a nest absconding response in these wasps, enhancing caracara predation. Some non-hymenopterous insects habitually living close to ant nests are also known to produce sulcatone as a possible means of avoiding ant predation. The rove beetles *Pella funestus* and *P. humeralis* excrete sulcatone from tergal glands when in the vicinity of *Lasius fuliginosus* nests. In these ants, sulcatone is used as a ‘panic-alarm’ inducing pheromone and thus when threatened the beetles release it, causing worker ants to disperse (Stoeffler et al. 2007). Sulcatone thus plays a critical role for foraging and predator avoidance in diverse a diverse range of insects.

All four *Gelis* species produced sulcatone but a close relative in the same subfamily (Cryptinae), *A. nens*, and a slightly more distant relative, *C. glomerata*, did not. Moreover, among the ants tested, sulcatone was detected in two *Lasius* species but not in *Myrmica rubra*. This suggests that the production of sulcatone is phylogenetically conserved in some *Gelis* and *Lasius* species and in some other (but not all) ant genera. The phylogenetic reconstruction showed the gelines to be monophyletic with *G. proximus* and *G. hortensis* being sister species, and *G. agilis* more distantly related. *Gelis areator* was placed among a more remote cluster of winged species, while *A. nens* and other closely related genera (e.g., *Lysibia*) form a more basal clade in the Cryptinae. Taking phylogeny into account, a plausible evolutionary trajectory in this clade is an early evolution of sulcatone production in the common ancestor of the gelines, as sulcatone is found in all *Gelis* species. Winglessness is confined to a subset of *Gelis* species (Schwarz and Shaw 1999), whereas other gelines as well as most other species in the Cryptinae are fully winged (Schwarz and Shaw 2000). Therefore, the most parsimonious scenario is that the common ancestor of *Gelis* species was fully winged, and that wings were lost later in the evolution of the gelines. This suggests that sulcatone evolved before winglessness evolved. Possibly, abdominal flexing and the ability to synthesize and secrete sulcatone were first employed as a means of defense against natural enemies which allowed the parasitoids to forage for hosts in habitats on the ground in which both ants and wolf spiders were also highly ubiquitous. Over time, wings may have been lost in some species to enhance an ant-like appearance beneficial for living on the ground and which augmented the benefits of the other ant-like traits.

Although our attention previously focused on chemical communication in *G. agilis* as a defense against predation by wolf spiders (Malcicka et al. 2015), it is important to stress visual cues may also be possible deterrents. Wolf spiders are known to have strong visual acuity and have excellent depth perception for objects at close range (Clemente et al. 2010; Land and Barth 1992). Furthermore, they detect differences in the spectrum of ultraviolet light (DeVoe 1972). The strong morphological resemblance of the wingless male gelines to ants may also be used as a cue by the spiders to avoid them. Interestingly, the spiders did not distinguish between *G. areator*, which secretes sulcatone, and *A. nens*, which does not. Both species were also much less susceptible to spider predation than *C. glomerata* in the choice assays. This suggests that *G. areator* may release lower concentrations of sulcatone than *G. proximus* and *G. hortensis* (notably it is not detected in olfactory assays with *G. aerator* but it is with *G. proximus*, *G. hortensis* and *G. agilis*). Furthermore, the strong similarity of *G. areator* with *A. nens* may make it hard for spiders to distinguish between the two species, even at close range. Alternatively, *A. nens* may secrete a different deterrent compound from the *Gelis* spp. that was not detected in our analyses.

One of the more contentious aspects in our understanding of the adaptive benefits of resemblance by one species or species group of another species or species group is whether this is a case of evolved mimicry or simply an example of trait convergence that may be incidental. Mimicry typically involves a tripartite interaction involving the ‘model’, the ‘mimic’ and the ‘operator’ (Dettner and Liepert 1994; Vane-Wright 1976). Two types of mimicry have been described: Batesian and Müllerian (Quicke 2017; Speed 1999). In the former, the mimic is a palatable species that resembles a toxic, unpalatable or dangerous species. The close resemblance of the mimic to the model fools potential predators or else invokes a flight response in them. In the case of Müllerian mimicry, a toxic species mimics another toxic species (Quicke 2017). Speed (1999) argued that some species may even exhibit characteristics of both mimicry strategies. In this instance we have a clear example of wingless gelines that share micro-habitats with ants that exhibit morphological similarity and which also produce sulcatone that is a known panic/alarm pheromone in several ants including the *Lasius* species studied here.

If wingless gelines are ant mimics, would argue that it appears to exhibit traits of both types (Batesian and Müllerian). However, as Keller et al. (2014) has pointed out, worker ants have economized their body structure to optimize the use of limited metabolic space for thoracic musculature. Thus, thoracic muscles are used in winged species both for flight and locomotion, resulting in a potential trade-off between different modes of activity. By contrast, in wingless species thoracic musculature can be used to for strengthening the jaws, neck, or be used exclusively for locomotion. One major difference between worker ants and female gelines, however, is that the latter must also mature eggs in order to reproduce; worker ants are sterile. Therefore, economizing on the thoracic musculature in wingless gelines may free other resources for egg production. In either of the above scenarios, the similarity in morphology of ants and gelines may simply be a form of convergent evolution rather than mimicry. On the other hand, this does not explain why wingless gelines produce the same repellent compound (sulcatone) as ants when they are agitated.

Myrmecomorphy, or ant mimicry, is well reported across a wide number of arthropod taxa (Cushing 2012; Mclver and Stonedahl 1993; Nelson et al. 2006). Other species are known to mimic morphological, behavioral, and chemical traits of ants, primarily as a means of defense. Many ants are rapacious predators and may kill a large number of other invertebrates to meet the nutritional demands of the growing larvae within the colony (Hölldobler and Wilson 1990). Moreover, they will aggressively defend their nests, including other workers, when they are attacked by other predators. Because of this, many ground-dwelling predators, such as spiders, habitually avoid ants when foraging (Oliveira 1988). Consequently, myrmecomorphy can be highly adaptive for other ground-dwelling arthropods living in the same local habitats as ants because other predators will avoid them.

This begs the question: is the morphological and chemical resemblance of wingless *Gelis* species to ants in some genera merely coincidental (e.g. a form of convergent evolution) or an example of evolved myrmecomorphy? At this stage it is difficult to say. As we explained earlier, the presence of eye-spots on the thorax of larvae or wings of adults of some species butterflies and moths (e.g. Papilionidae, Saturnidae, Sphingidae) has been explained as both a form of anti-predatory mimicry of the eyes of snakes (Hossie and Sherratt 2012) but also as a non-mimicking consequence of sexual selection (Monteiro 2015; Oliver et al. 2009). Given the abundance of ants in many habitats and the important role they play in many ground habitats, we believe that the expression of several traits in wingless *Gelis* species that resemble ants may indeed be an example of multi-trait or multimodal mimicry (Rowe and Guilford 1999; Rowe and Halpin 2013). However, more studies are needed incorporating phylogeny, behavior and ecology to determine if gelines mimic ants and if so what kind of mimcry they exhibit.
